# *Dictamnus dasycarpus* Turcz. Root Bark Improves Skin Barrier Function and Symptoms of Atopic Dermatitis in Mice

**DOI:** 10.3390/ijms252313178

**Published:** 2024-12-07

**Authors:** Sangjun Park, Jinkyu Yang, Kyoungmin Sun, Seonah Park, Jimi Lee, Soyeon Kim, Ji Hyo Lyu, Hyungwoo Kim

**Affiliations:** 1 Division of Pharmacology, School of Korean Medicine, Pusan National University, Yangsan 50612, Republic of Korea; sjpark177@gmail.com (S.P.); vo2mxlift@gmail.com (J.Y.); abd88888@naver.com (K.S.); alice9912@naver.com (S.P.); super933@naver.com (J.L.); amugdale@pusan.ac.kr (S.K.); 2 Herbal Medicine Resources Research Center, Korea Institute of Oriental Medicine, Naju 58245, Republic of Korea

**Keywords:** *Dictamus dasycarpus*, inflammation, skin barrier function, atopic dermatitis, skincare

## Abstract

The root bark of *Dictamus dasycarpus* Turcz. has been traditionally used for the topical treatment of skin disorders like pruritus. This study was designed to investigate the inflammatory and skin barrier protective effects of *D. dasycarpus* in mice with calcipotriol (MC903)-induced atopic dermatitis (AD). Topical skin lesions on male Balb/c mice (8 weeks old) were treated topically with an ethanolic extract of *D. dasycarpus* (EEDD), and skin water content, water holding capacity (WHC), histopathological abnormalities, and inflammatory cytokine and chemokine levels were investigated. Topical application of EEDD effectively alleviated skin lesion severity, improved skin water content and WHC, and ameliorated histopathological abnormalities, including hyperkeratosis, blood vessel numbers near the epidermis, spongiotic changes, and immune cell infiltration in skin tissues. EEDD also suppressed inflammatory cytokines and chemokines, such as tumor necrosis factor (TNF)-α, thymic stromal lymphopoietin (TSLP), interleukin (IL)-1β, IL-4, IL-8, and monocyte chemotactic protein (MCP)-1. In RAW264.7 cells, EEDD reduced nuclear factor kappa-light-chain-enhancer of activated B cells (NF-kB) expression and suppressed the phosphorylations of extracellular signal-regulated kinase (ERK) and p38. These results suggest that the root bark of *D. dasycarpus* has therapeutic potential due to its anti-dermatitis and skin barrier protective effects in AD and that it could be used as an ingredient in skincare products.

## 1. Introduction

Atopic dermatitis (AD) is a chronic inflammatory skin disease with symptoms of dryness, flaking, itchiness, and erythema. AD exhibits chronic characteristics as symptoms recur in cycles of improvement and relapse due to its complicated etiology, which includes genetic and environmental factors, immunological abnormalities, and skin barrier dysfunction [1].

A recent study demonstrated a strong connection between AD severity and skin barrier function [2]. Furthermore, a mutation in filaggrin, an essential component of the stratum corneum (SC), was found to specifically induce inflammatory reactions by damaging skin barrier function and simultaneously creating an environment conducive to recurrent inflammatory response [3]. AD impairs skin barrier function and reduces its ability to retain moisture, which leads to flaky skin and aggravation of hyperkeratosis and inflammation [4].

Thymic stromal lymphopoietin (TSLP) is known to increase the severity of AD by stimulating CD11c+ myeloid dendritic cells and thus inducing Th2-mediated inflammation [5], downregulating filaggrin expression levels [6], and causing inflammatory response by T cell migration in filaggrin-deficient skin [7]. TSLP levels have been reported to significantly correlate with AD severity, and as a result, a new approach based on targeting TSLP has been adopted to prevent AD aggravation [8].

Steroid therapy is usually used initially to treat AD. However, the widespread public fear of topical steroids and the high prevalence of AD in children create practical difficulties for caregivers regarding decision-making on long-term topical steroid treatment [9]. This situation demonstrates a need for an alternative topical treatment for AD.

*Dictamnus dasycarpus* Turcz. belongs to the *Rutaceae* family and is a traditional herbal medicine characterized by a bitter taste and cold properties. This perennial herb is commonly found throughout Northeast Asia, including Korea. In traditional medicine, *D. dasycarpus* is commonly utilized in topical applications to reduce fever and enhance blood circulation in the skin. In particular, the root bark of *D. dasycarpus* has long been utilized in traditional medicine to alleviate conditions such as pruritus, eczema, asthma, arthralgia, and jaundice [10].

Recently, a number of chemical compounds have been isolated from *D. dasycarpus*, including quinoline alkaloids, limonoids, sesquiterpenes, and flavonoids. Furthermore, studies have shown its pharmacological activities include anti-inflammatory, anti-fungal, anti-allergic, anti-oxidative, and cardiovascular protective effects [11]. Our laboratory, which is conducting ongoing research on *D. dasycarpus*, has demonstrated that its root bark extract suppresses keratinocyte proliferation and has anti-inflammatory effects on contact dermatitis (CD) due to regulations of ICAM-1, IFN-γ, and TNF-α [12,13]. Nevertheless, its effects on inflammatory responses and skin barrier function in AD have not been investigated.

This study was undertaken to investigate the effects of *D. dasycarpus* root bark extract on skin barrier functional recovery and inflammation in an MC903 (calcipotriol, a vitamin D3 analogue with low calcemic activity) induced model of AD by measuring inflammatory cytokine levels, including those of TSLP and water retention capacities, performing histopathological analysis, and scoring skin lesion severity.

## 2. Results

### 2.1. EEDD Alleviated Skin Lesions and Prevented Increases in Erythema Index, Skin Thickness, and Weight

**Figure 1:**
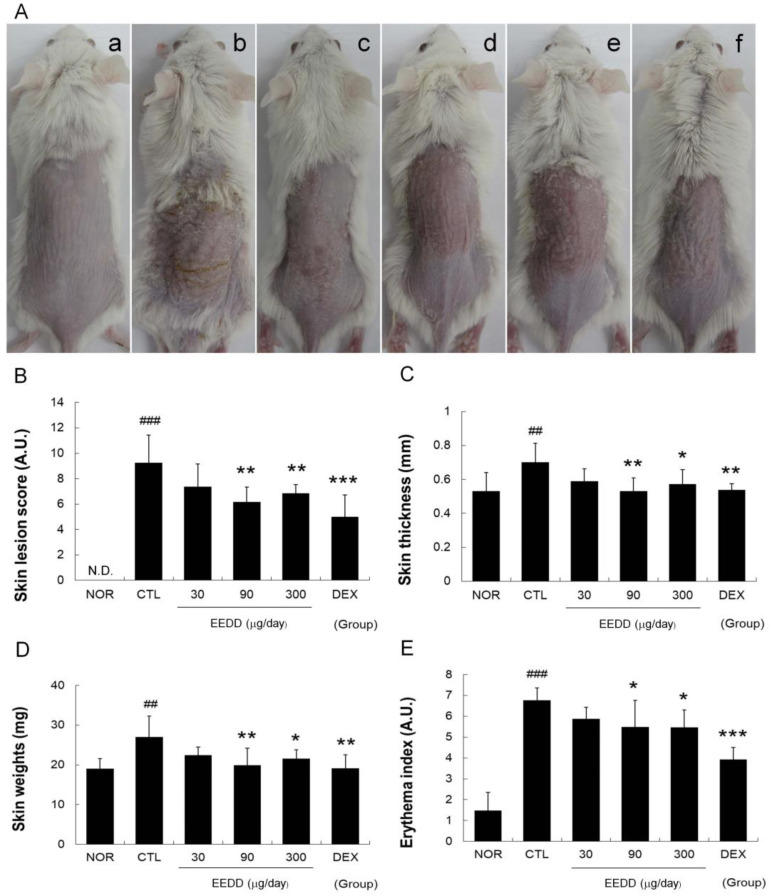
Effects of EEDD on skin color and lesions in AD mice (**A**) a, treatment-naïve mice (NOR); b, AD control (CTL); c, 30 μg/day EEDD; d, 90 μg/day EEDD; e, 300 μg/day EEDD; f, 150 μg/day DEX. (**B**) Skin lesion scores; (**C**) Cutaneous thickness; (**D**) Weight of skin samples; (**E**) Erythema indices. A.U., arbitrary units; N.D., undetectable; EEDD, ethanol extract of *D. dasycarpus* root bark; DEX, dexamethasone. Results are presented as means ± SDs. ## *p* < 0.01 and ### *p* < 0.001 vs. NOR; * *p* < 0.05, ** *p* < 0.01, and *** *p* < 0.001 vs. CTL.

Repeated applications of MC903 to dorsal skin caused skin sclerosis, fissures, flaking, and erythema. However, treatment with EEDD for six consecutive days alleviated these symptoms (Figure 1A). EEDD (at 90 or 300 μg/day) and DEX significantly reduced severity scores and erythema indices compared to the CTL group (Figure 1B,E).

MC903 increased cutaneous thickness and weight of skin samples in the CTL group, and these increases were prevented by topical EEDD (90 or 300 μg/day) (Figure 1C,D).

### 2.2. EEDD Enhanced Skin Water Content and Water-Holding Capacity

**Figure 2:**
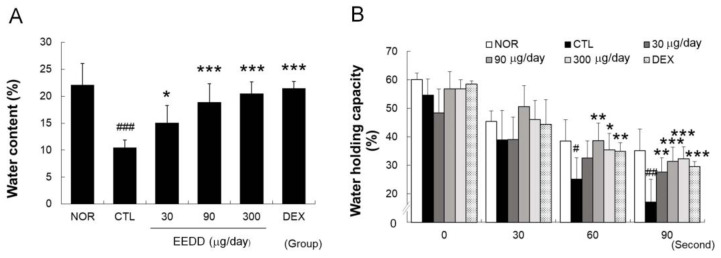
Effects of EEDD on skin water contents and WHCs in AD mice. (**A**) Water content; (**B**) WHC. EEDD, ethanol extract of *D. dasycarpus*, root bark; DEX, dexamethasone. Results are presented as means ± SDs. # *p* < 0.05, ## *p* < 0.01, and ### *p* < 0.001 vs. NOR; * *p* < 0.05, ** *p* < 0.01, and *** *p* < 0.001 vs. CTL.

Water content in the CTL group decreased to nearly half that in the NOR group, and topical application of EEDD significantly increased water contents (Figure 2A). WHC decreased in the CTL group to ~one-third of baseline levels at 90 s. Significant improvements in WHC were observed in the EEDD (90 and 300 μg/day) and DEX groups (Figure 2B).

### 2.3. EEDD Inhibited Histopathological Defects in Inflamed Tissues

**Figure 3:**
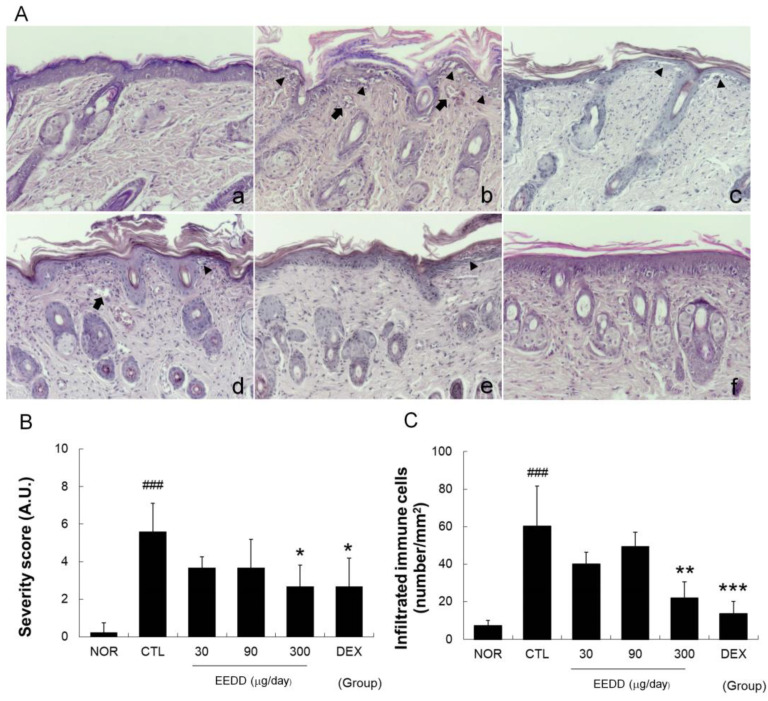
Effects of EEDD on histopathological abnormalities in inflamed tissues (**A**) Abbreviations are consistent with those in Figure 1A. The solid arrow shows a blood vessel located near the epidermis, and the solid wedge indicates an indistinct basal layer between the epidermis and dermis (100×); (**B**) Severity scores; (**C**) Infiltrating immune cells. EEDD, ethanol extract of *D. dasycarpus*, root bark; DEX, dexamethasone. Results are presented as means ± SDs. ### *p* < 0.001 vs. the NOR; * *p* < 0.05, ** *p* < 0.01, and *** *p* < 0.001 vs. CTL.

Topical application of MC903 for 8 days induced superficial blood vessel formation near the epidermis, hyperkeratosis, spongiotic changes, and immune cell infiltration and disrupted the basal layer (Figure 3A). MC903 also markedly increased immune cell infiltration and severity scores in the CTL group, whereas EEDD (300 μg/day) and DEX significantly decreased immune cell infiltration and severity scores (Figure 3B,C).

### 2.4. EEDD Prevented MC903-Induced Increases in TNF-α, IL-2, IL-4, and IL-6 in Inflamed Tissues

**Figure 4:**
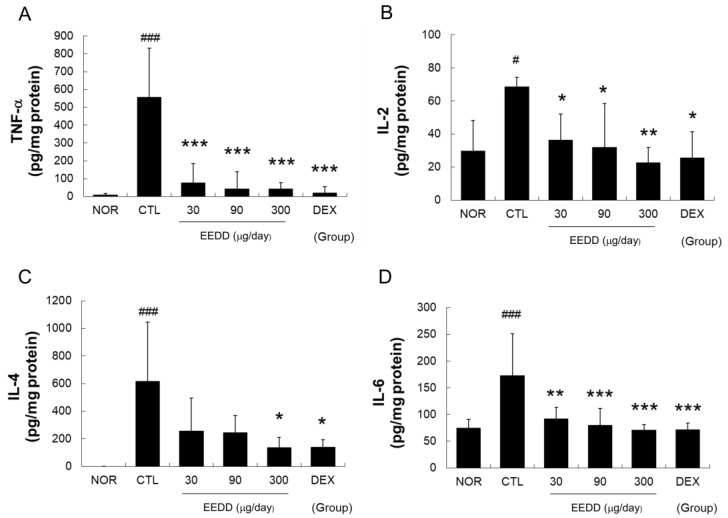
Effects of EEDD on MC903-induced TNF-α, IL-2, IL-4, and IL-6 increases in skin tissues (**A**), TNF-α; (**B**), IL-2; (**C**), IL-4; (**D**), IL-6. EEDD, ethanol extract of *D. dasycarpus*, root bark; DEX, dexamethasone. Results are presented as means ± SDs. # *p* < 0.05 and ### *p* < 0.001 vs. the NOR; * *p* < 0.05, ** *p* < 0.01, and *** *p* < 0.001 vs. CTL.

Cytokine (TNF-α, IL-4, IL-6, and IL-2) levels were significantly elevated in the CTL group. TNF-α, IL-2, and IL-6 levels were significantly lowered by EEDD at all concentrations versus the CTL group (Figure 4A,B,D), and IL-4 levels were significantly lowered by EEDD at 300 μg/day (Figure 4C). DEX significantly reduced all four cytokine levels (Figure 4).

### 2.5. EEDD Suppressed the Expressions of TSLP and Inflammatory Factors

**Figure 5:**
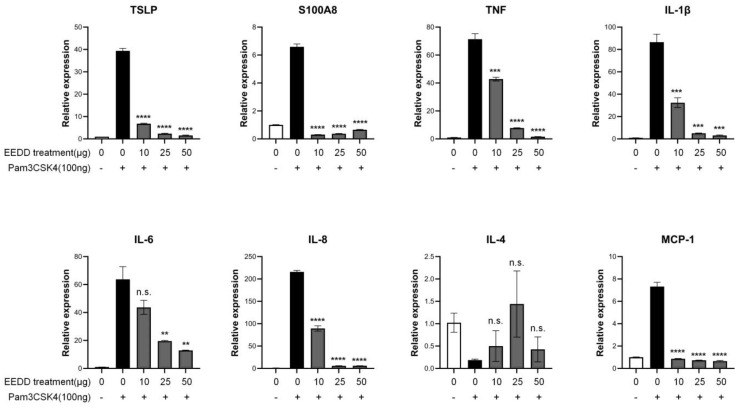
Effects of EEDD on the mRNA levels of cytokines and chemokines in RAW264.7 cells. Levels of TSLP, S100A8, TNF, IL-1β, IL-6, IL-8, IL-4, and MCP-1 were measured by quantitative PCR. Results are presented as means ± SDs. n.s., not significant; ** *p* < 0.01, *** *p* < 0.001, and **** *p* < 0.0001 vs. CTL.

In RAW264.7 cells, the TLR2/TLR1 agonist Pam3CysSerLys4 (Pam3CSK4) increased the expressions of TSLP and S100A8, and both increases were significantly reduced by EEDD. In addition, Pam3CSK4-induced increases in TNF, IL-1β, IL-6, IL-8, and monocyte chemoattractant protein (MCP)-1 levels were also significantly decreased by EEDD (Figure 5).

### 2.6. EEDD Regulated NF-κB (p65) Translocation and the MAPK Pathway

**Figure 6:**
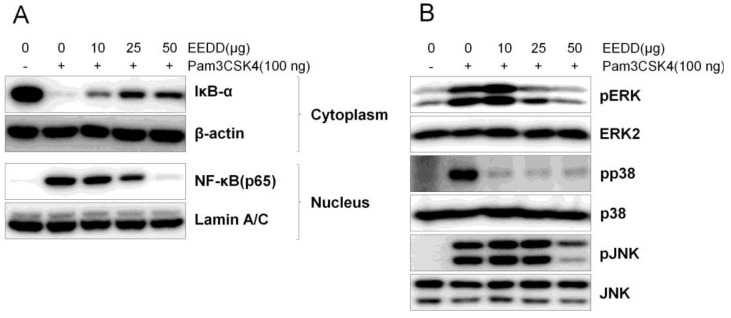
Effects of EEDD on NF-κB and MAPK signalling pathways in RAW264.7 cells. The protein levels of IκB-α, NF-κB (**A**), ERK, p38, and JNK (**B**) were determined by Western blot.

Cytoplasmic IκB-α was degraded by Pam3CSK4 in RAW264.7 cells, but this was prevented by EEDD treatment. Consistent with this observation, the Pam3CSK4-induced nuclear translocation of NF-κB (p65) was decreased dose-dependently by EEDD (Figure 6A). Additionally, Pam3CSK4-induced phosphorylation of ERK and p38 was reduced by EEDD, whereas the Pam3CSK4-induced phosphorylation of JNK was not reduced (Figure 6B).

## 3. Discussion

The beauty market attracts considerable attention in modern society. Proper skin moisture is essential for beauty, and rough skin is closely related to moisture deficiency. It has been demonstrated that high levels of hydration help keep skin smoother and reduce wrinkling caused by rough skin [14]. This is because the outermost layer of skin, the SC, retains moisture and protects the skin from external stimuli [15].

Skin barrier function impairment is closely associated with the worsening of AD symptoms [4]. Elevated trans-epidermal water loss (TEWL) and low SC hydration are commonly observed in AD [4], and a deficiency of filaggrin, a major natural moisturizing factor, in AD reduces WHC and SC structural integrity, leading to increased water loss [16]. As a result, skin becomes dry and sensitive to external stimuli, which in turn triggers pruritus [17].

TSLP, which is overexpressed in keratinocytes, is a cytokine that promotes Th2 immune response, leading to tissue disruption and skin barrier dysfunction [18]. TSLP is also induced by allergens and skin barrier defects in AD [19], and as a result, the epidermis becomes more vulnerable to external stimuli and immune responses, which in turn exacerbates the symptoms of AD. TSLP is a key molecule in AD, and the development of drugs targeting TSLP, such as tezepelumab, is currently in progress [8].

In our study, EEDD significantly reduced skin lesion severity scores, particularly for scale, and improved erythema levels (Figure 1A,B,E). Additionally, EEDD reduced MC903-induced increases in the weight of skin samples and cutaneous thickness (Figure 1C,D). It would appear that EEDD augments the maintenance of epidermal homeostasis by promoting SC desquamation and suppressing inflammatory response. These findings demonstrate that EEDD meaningfully alleviates superficial symptoms in AD and has potential value as a topical treatment.

As shown in Figure 2, MC903 induced epidermal water loss and WHC impairment, indicating skin barrier dysfunction, and treatment with EEDD effectively mitigated the decrease in skin water content and prevented WHC impairment in a dose-dependent manner. These observations show that EEDD prevented MC903-induced skin barrier dysfunction.

Pro-inflammatory cytokines produced by inflammation activate vascular endothelial growth factor and increase blood vessel numbers near the epidermis [20]. Blood vessels are more actively produced during chronic inflammation, and this process contributes to AD symptoms by sustaining inflammatory response through blood flow increase [21]. We found EEDD inhibited hyperkeratosis, the production of superficial blood vessels, and basal layer disruption in inflamed tissues. In addition, it also effectively prevented immune cell infiltration (Figure 3). These results indicate that EEDD can ameliorate the histopathological abnormalities commonly observed in AD patients.

Hyperkeratosis refers to thickening of the outer skin layer due to desquamation abnormalities and causes SC cells to clump together and form large scales or flakes [22]. Our results showed EEDD reduced hyperkeratosis (Figure 3) and scab formation on skin (Figure 1), which were interpreted as alleviation of the superficial symptoms of AD by decreasing hyperkeratosis. It is also possible that the increase in blood vessel numbers in epidermis contributed to observed increases in erythema indices.

Basal layer disruption can lead to physical tissue damage, such as fissures, which are likely to have a direct negative impact on skin barrier function. EEDD reduced MC903-induced basal layer disruption (Figure 3), physical tissue damage (Figure 1), and skin barrier dysfunction (Figure 2). These results indicate that EEDD prevented physical tissue damage, such as disruption of the basal layer, as demonstrated by a significant reduction in skin lesion scores (Figure 1). Furthermore, by preventing physical skin damage, EEDD effectively improved skin barrier dysfunction.

TNF-α contributes to early skin inflammation and activates keratinocytes, and thus, causes epidermal hyper-proliferation [23], and IL-1β amplifies inflammatory response and promotes hyperkeratosis [24]. EEDD significantly reduced the production of TNF-α and IL-1β (Figure 4 and Figure 5) in a dose-dependent manner, indicating reduced keratinocyte stimulation. By lowering levels of these pro-inflammatory cytokines, EEDD effectively decreased MC903-induced increases in cutaneous thickness by inhibiting epidermal hyper-proliferation and reduced scab formation by preventing hyperkeratosis.

IL-4 promotes allergic skin inflammation by stimulating the production of IgE and recruiting eosinophils and plays an important role in Th2 cell differentiation [25]. IL-2 is also associated with T-cell proliferation and indirectly supports Th2 cell differentiation [26]. Similarly, TSLP induces Th2-type immune responses via dendritic cells and mast cells [8], and S100A8, a member of the S100 protein family, induces inflammatory cytokines, leading to sustained immune responses [26]. A Th2-skewed immune reaction is a classical pathogenic mechanism in AD. Th2 immune responses trigger inflammation and lead to skin tissue disruption, resulting in the surface symptoms of AD. We found that EEDD mitigated tissue damage (Figure 3) and lowered IL-4, TSLP, S100A8, and IL-2 levels (Figure 4 and Figure 5). These results imply that EEDD might improve the histopathological abnormalities of AD by inhibiting the Th2-skewed immune reaction.

It is well established that TSLP overexpression results in skin barrier dysfunction [19] and that IL-4 weakens the skin barrier and promotes skin dryness and water loss [27]. However, EEDD effectively prevented water loss and improved WHC (Figure 2), which suggests that the protective effect of EEDD on skin barrier dysfunction might be related to the downregulations of TSLP and IL-4.

IL-6 contributes to chronic inflammation by promoting the infiltration of T cells and neutrophils [25]. IL-8 (C-X-C ligand 8, CXCL8) is a chemokine that interacts specifically with the CXCR1 and CXCR2 receptors on immune cells, facilitating their migration to sites of inflammation [28]. MCP-1 is a chemokine that is upregulated in AD by pro-inflammatory cytokines such as TNF-α, IL-1β, and IL-6 [29]. We found that EEDD effectively inhibited immune cell infiltration (Figure 3), which was probably related to the downregulations of IL-6, IL-8, and MCP-1 (Figure 4 and Figure 5) in a dose-dependent manner. These findings suggest that EEDD inhibits immune cell infiltration and suppresses the progression of inflammation by modulating the expressions of these chemokines.

The NF-κB pathway plays a crucial role in the proliferation of keratinocytes. It is also important for the induction of chemokines and cytokines [30]. The MAPK pathway also plays an important role in cytokine and chemokine expression [31]. According to our results, EEDD effectively inhibited NF-κB expression and the phosphorylations of ERK and p38, implying that EEDD suppressed the production of cytokines and chemokines by inhibiting NF-κB, ERK, and p38 pathways. In addition, we found in our previous studies that the methanol extract of *D. dasycarpus* inhibited ICAM-1 expression via the NF-κB pathway in immortalized human keratinocytes [12]. This concurs with the present study and indicates that the inhibition of ICAM-1 expression through the NF-κB pathway contributed to reduced immune cell infiltration (Figure 3).

Typically, a healthy epidermis is thick and elastic because as the SC thickens, the skin barrier function is enhanced, and the skin can better prevent TEWL [32]. However, in AD, an increase in cutaneous thickness is used to prevent excessive water loss [33]. An increase in cutaneous thickness was observed in the CTL group, reflecting this compensatory mechanism. In the present study, EEDD reduced cutaneous thickness but increased skin water content and WHC (Figure 1C and Figure 2). Considering the aforementioned results, it appears that EEDD reduced cutaneous thickness by inhibiting the compensatory mechanism associated with water loss.

Corticosteroids have been shown to induce splenic atrophy [34], and we observed this effect but not in the EEDD groups (Supplementary Data S1, Figure S1). This indicates that EEDD did not cause systemic immune suppression and suggests its anti-inflammatory effects were due to some mechanism other than one involving corticosteroids.

While many botanical extracts have the potential to cause allergic reactions and photosensitization, there is a concern as a topical treatment that *D. dasycarpus* could induce allergic contact dermatitis. However, *D. dasycarpus* has not been reported to cause skin-related adverse reactions [11]. Furthermore, it has been demonstrated to exhibit anti-inflammatory effects on allergic contact dermatitis [13].

Taken together, these results demonstrate that the root bark of *D. dasycarpus* has potential use as an AD treatment or as an active ingredient in skincare products due to its anti-inflammatory effects and skin barrier-repairing effects elicited by the regulations of cytokines and chemokines.

## 4. Materials and Methods

### 4.1. Preparation of the Dictamnus Dasycarpus Root Bark Extract

*D. dasycarpus* root bark was obtained from the Kwangmyungdang Drug Company (Ulsan, Republic of Korea). Briefly, dried root bark (100 g) was soaked in 500 mL of ethanol, sonicated for 5 min, and extracted for 24 h. The supernatant was collected, and the root bark was subjected to a second extraction using an additional 500 mL of ethanol for another 24 h with a 5-min sonication. The extract was then filtered through Whatman No. 20 filter paper, concentrated using a rotary evaporator (Eyela, Tokyo, Japan), and freeze-dried (Labconco, Kansas City, MO, USA). This process yielded 14.32 g of lyophilized extract (14.32% yield). This ethanol extract of *D. dasycarpus* (EEDD, voucher No. MS2021-1029) was stored at the Division of Pharmacology, School of Korean Medicine, Pusan National University (Supplementary Data S2, Figure S2).

### 4.2. Animals

The animal study was conducted using 8-week-old male Balb/c mice sourced from Hana Biotech (Gyeonggi, Republic of Korea). The mice were housed in a specific pathogen-free facility under a 12-h light/dark cycle and given unrestricted access to standard rodent chow and water. All experimental procedures were approved by and complied with the guidelines issued by the Animal Care and Use Committee of Pusan National University (Approval No. PNU-2022-0195).

### 4.3. Induction of AD and Experimental Design

AD was induced as we previously described [35]. Animals were randomly allocated to six groups, viz. the treatment-naïve group (NOR group, n = 6), the AD control group (the AD CTL group, n = 8), three EEDD treatment groups (n = 8), or a dexamethasone positive control group (the dexamethasone (DEX) group, n = 8). The backs of animals, except animals in the NOR group, were sensitized for 3 consecutive days with MC903 (0.1 mM), and all animal backs were then shaved (day 4). Dorsal skins in the AD CTL, the EEDD treatment groups, and the DEX group were challenged with MC903 (4 nM/day) for 8 consecutive days (days 7–14), and animals in the EEDD treatment groups and the DEX group were treated with EEDD at 30, 90, or 300 µg/day, or DEX at 150 μg/day for 6 consecutive days (days 9 to 14). The experimental design is detailed in Supplementary Materials (Supplementary Data S3, Figure S3).

### 4.4. Assessment of Skin Lesion Severities

The severities of skin lesions, viz. erythema, scale, induration, and fissure formation, were assessed at the end of the experiment (day 15) using a 4-point scale (0 (none), 1 (mild), 2 (moderate), and 3 (severe)).

### 4.5. Measurement of Skin Thicknesses, Weight, and Color

At the end of the experiment (day 15), cutaneous thickness was measured using a Vernier calipers (Mitutoyo, Tokyo, Japan), and the weight of skin samples was measured using a biopsy punch (5 mm diameter). Erythema and melanin levels were assessed at three different sites per mouse using a skin colorimeter (DSM II, Cortex Technology, Horsens, Denmark), and the average of these three measurements was used to evaluate overall skin color [35].

### 4.6. Skin Water Content and Water-Holding Capacity

Skin water content and water-holding capacity (WHC) were evaluated using a skin hygrometer (Scalar Corporation, Tokyo, Japan), as we previously described [35]. Briefly, skin water contents were assessed at three dorsal locations per mouse. WHC was measured immediately after removing a wet gauze (1 × 1 cm, soaked in distilled water) placed on a shaved back for 30 s. WHC measurements were taken four times at the same locations, and results were averaged.

### 4.7. Histopathological Examination

Inflamed tissues were resected, embedded in paraffin, and stained with hematoxylin and eosin (H&E) for histopathological examination. These tissues were then examined under an optical microscope (at 100×) to evaluate superficial blood vessel numbers, hyperkeratosis, and spongiotic changes. Numbers of infiltrating immune cells were determined as we previously described [35].

### 4.8. Cytokine Levels

Cytokine levels in skin samples were measured using a bead array mouse inflammation kit (BD, San Jose, CA, USA) [35]. The resected dorsal skin tissues were lysed and homogenized using a protein extraction buffer (Thermo Scientific, Mount Prospect, IL, USA). Following the procedure, 50 μg of each lysate was utilized to evaluate the levels of TNF-α, IL-2, IL-4, and IL-6. The detection limits for TNF-α, IL-2, IL-4, and IL-6 are 0.9, 0.1, 0.03, and 1.4 pg/mL, respectively.

### 4.9. Cell Culture

RAW264.7 cells (a murine macrophage cell line) were purchased from the ATCC (American Type Culture Collection, Rockville, MD, USA) and cultured in Dulbecco’s Modified Eagle’s Medium (DMEM) supplemented with 200 mg/L L-glutamine (Hyclone, Logan, UT, USA), 10% (*v*/*v*) heat-inactivated fetal bovine serum (FBS) (Invitrogen, Carlsbad, CA, USA), and 100 U/mL penicillin and 100 μg/mL streptomycin. Cells were maintained in a humidified incubator at 5% CO_2_ prior to experiments.

### 4.10. Isolation of Total RNA and RT-qPCR

Total RNA was isolated from cultured cells using TRIzol reagent (Invitrogen, Carlsbad, CA, USA), and cDNA was synthesized as previously described [36]. Quantitative PCR (qPCR) was conducted utilizing a Rotor-Gene Q system (Qiagen, Hilden, NRW, Germany) using a TOPreal SYBR Green qPCR premix (Enzynomics, Daejeon, Republic of Korea). GAPDH was used as the internal control, and Ct values were determined using the 2−ΔΔCt formula for accurate quantitative analysis. Target primers used for qPCR are described in Supplementary Materials (Supplementary Data S4, Table S1).

### 4.11. Western Blot Analysis

Protein extraction and Western blot were conducted as previously described [36]. Briefly, total cells and nuclear proteins were extracted and quantified using the Bradford method. Equal amounts of protein extracts were separated by SDS-PAGE and transferred to a PVDF membrane. After blocking the membrane with 5% non-fat dry milk, it was incubated overnight with primary antibodies targeting proteins like IκB-α, NF-κB (p65), ERK2, p-ERK, p-p38, p38, p-JNK, JNK, β-actin, and lamin A/C. The membrane was then incubated for 1 h at room temperature with HRP-conjugated secondary antibodies for detection. The blots were visualized using an enhanced chemiluminescence system (SuperSignal^®^ West Femto, Thermo Scientific, Mount Prospect, IL, USA).

### 4.12. Statistical Analysis

Data were analyzed using one-way ANOVA followed by Dunnett’s post hoc test for multiple comparisons. The statistical analysis was performed using GraphPad Prism 5 for Windows, version 5.01 (GraphPad Software Inc., La Jolla, CA, USA). The results are expressed as means ± standard deviations (SDs), and statistical significance was accepted for *p* values < 0.05.

## 5. Conclusions

In this study, EEDD was found to suppress the activations of NF-κB, ERK, and p38 pathways, which in turn reduced the production of pro-inflammatory cytokines (TNF-α and IL-1β), Th2-skewing cytokines (IL-4, IL-2, TSLP, and S100A8), and chemokines (IL-8, IL-6, and MCP-1) in RAW264.7 macrophages. Furthermore, EEDD inhibited immune cell infiltration into the epidermis and prevented histological abnormalities, including the production of superficial blood vessels and hyperkeratosis in male Balb/c mice. EEDD also effectively restored MC903-induced skin barrier dysfunction and improved AD-like skin lesions. Overall, these results suggest that the root bark of *D. dasycarpus* has therapeutic potential in AD and offers a possible alternative to corticosteroids. In addition, we suggest it could be used as a functional ingredient in skincare products due to its ability to improve skin lesion symptoms and enhance skin barrier function.

## Data Availability

The data presented in this study are available upon request from the corresponding author.

## Supplementary Materials

The following supporting information can be downloaded at: https://www.mdpi.com/article/10.3390/ijms252313178/s1.
