# Supplementary material for: Dictamnus dasycarpus Turcz. Root Bark Improves Skin Barrier Function and Symptoms of Atopic Dermatitis in Mice
Source: Int J Mol Sci. 2024 Dec 7;25(23):13178. doi: 10.3390/ijms252313178 (PMC11641830; doi:10.3390/ijms252313178)
Supplement: Supplementary file 1 [file ijms-25-13178-s001.zip › Supplementary data S1. Spleen body weight ratio.pdf]

Supplementary data S1

Measurement of body and spleen weights

Body weights of each mouse were measured on day 1 and day 15 using electronic scale (CAS, Gyeonggi, Korea) respectively. Changes in body weights were expressed as percentages of weight on day 1. Spleen weights were measured on day 15 using microbalance (Sartorius, Gyeonggi, Korea). The effects of EEDD on spleen weights are presented as the spleen body weight ratio.

EEDD did not affect spleen enlargement.

The effect of EEDD on spleen enlargement was estimated by determining spleen/body weight ratio. The spleen/body weight ratios in the EEDD treated groups were similar to that of the CTL group. DEX treatment lowered spleen body weight ratio significantly (Figure S2).

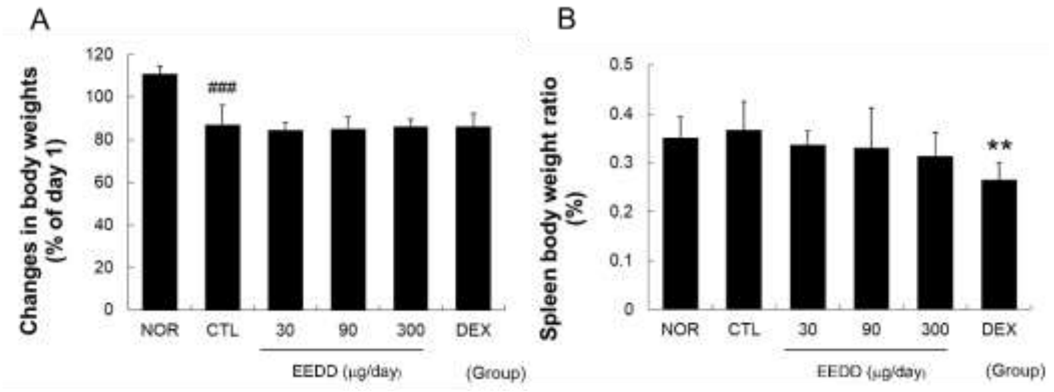

Figure S1. Effects of EEDD on spleen/body weight ratio in AD mice. Body and spleen weight were measured on day 15 and the spleen/body weight ratio was calculated. (A), changes in body weights; (B), spleen/body weight ratio. EEDD, ethanol extract of *D. dasycarpus*, root bark; DEX, dexamethasone. $^{###}P < 0.001$ vs. NOR; $^{**}P < 0.01$ vs. CTL.
