# Supplementary material for: Dictamnus dasycarpus Turcz. Root Bark Improves Skin Barrier Function and Symptoms of Atopic Dermatitis in Mice
Source: Int J Mol Sci. 2024 Dec 7;25(23):13178. doi: 10.3390/ijms252313178 (PMC11641830; doi:10.3390/ijms252313178)
Supplement: Supplementary file 1 [file ijms-25-13178-s001.zip › Supplementary data S2. Finger print data.pdf]

Supplementary data S2

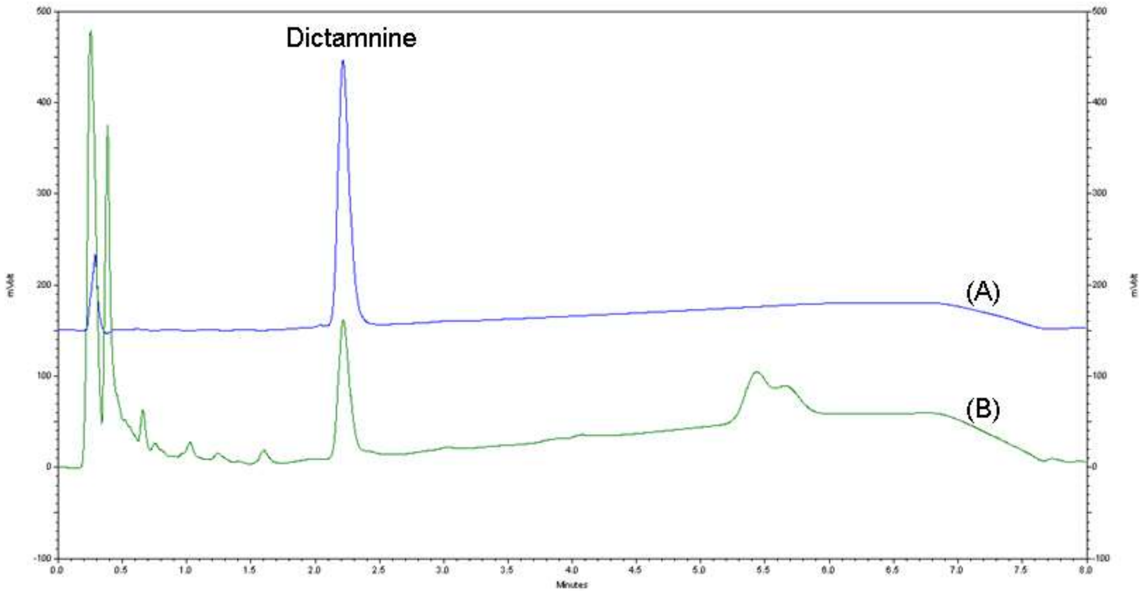

Figure S2. Chromatograms for Dictamnine (A) and root bark of *Dictamus dasycarpus* Turcz. (B) at UV wavelength of 235 nm.

Identification of dictamine in extract of *D. dasycarpus* root bark

The peak of dictamine was detected in the retention time of 2.218 min and it was also observed in *D. dasycarpus* extract with same retention time. Dictamine in *D. dasycarpus* extract was found as largest peak excluding two peak eluting within the retention time of 2.218 min as shown in Figure S2.

Preparation of standard solution

One milligram of dictamnine was accurately weighed and dissolved in methanol at the concentration of 100 μg/mL and the solution was 10-fold diluted before the injection.

Chromatographic conditions

Smart LC system comprised a LC800 (GL sciences, Japan) equipped with built-in apparatus including solvent delivery unit, autosampler, column oven and UV-visible detector. The acquired data was processed using EZChrom Elite software (Ver. 3.3.2 SP1). Chromatographic separation was performed on a Inertsil ODS-4 column (2.1 x 50 mm, 2 μm; GL sciences, Japan) with the temperature at 35 °C. The mobile phase consisted of water (A) and acetonitrile (B). A gradient program of mobile phase was used as follows: 5% (B) maintained for 5 min, 5–90% (B) over 5–7 min. The flow rate was set at 0.4 mL/min and the injection volume was 1 μL. Detection wavelength of dictamine was set at 235 nm.
