# Supplementary material for: Dictamnus dasycarpus Turcz. Root Bark Improves Skin Barrier Function and Symptoms of Atopic Dermatitis in Mice
Source: Int J Mol Sci. 2024 Dec 7;25(23):13178. doi: 10.3390/ijms252313178 (PMC11641830; doi:10.3390/ijms252313178)
Supplement: Supplementary file 1 [file ijms-25-13178-s001.zip › Supplementary data S3. Experimental design.pdf]

Supplementary data S3

Animals were randomly allocated to six groups, viz. the treatment-naïve group (NOR group, n=6), the AD control group (the AD CTL group, n=8), three EEDD treatment groups (n=8), or a dexamethasone positive control group (the dexamethasone (DEX) group, n=8). The backs of animals, except animals in the NOR group, were sensitized for 3 consecutive days with MC903 (0.1 mM), and all animal backs were then shaved (day 4). Dorsal skins in the AD CTL, the EEDD treatment groups, and the DEX group were challenged with MC903 (4 nM/day) for 8 consecutive days (days 7-14), and animals in the EEDD treatment groups and the DEX group were treated with EEDD at 30, 90, or 300 μg/day, or DEX at 150 μg/day for 6 consecutive days (days 9 to 14). The experimental design is detailed in Figure S1.

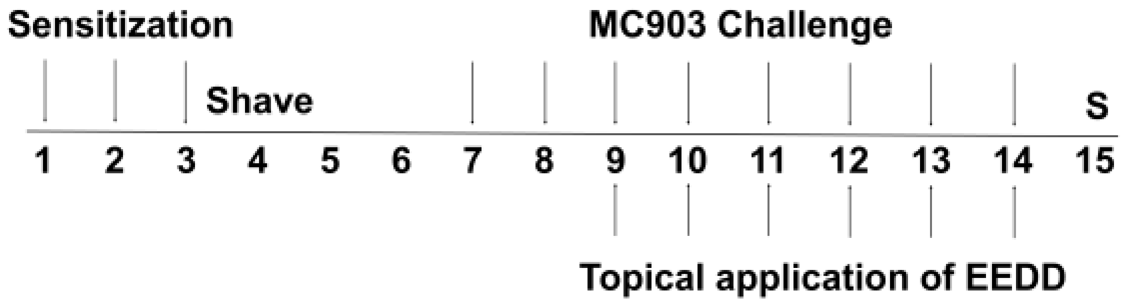

Figure S3. Experimental schedule. EEDD means ethanol extract of *D. dasycarpus* root bark, S means sacrifice.
