# Supplementary material for: Dictamnus dasycarpus Turcz. Root Bark Improves Skin Barrier Function and Symptoms of Atopic Dermatitis in Mice
Source: Int J Mol Sci. 2024 Dec 7;25(23):13178. doi: 10.3390/ijms252313178 (PMC11641830; doi:10.3390/ijms252313178)
Supplement: Supplementary file 1 [file ijms-25-13178-s001.zip › Supplementary data S4. The primer sets for qPCR.pdf]

Supplementary data S4

Table S1. Target primers used for qPCR in this study

Target gene	Primer sequences (5' to 3' direction)
TNF-α	Forward: CTCTCATCAGTTCTATGGCC<br>Reverse: TTGAAGAGAACCTGGGAGTA
IL-1β	Forward: CAGGCAGTATCACTCATTGT<br>Reverse: CCAGCAGGTTATCATCATCA
IL-4	Forward: GAGAGAGATCATCGGCATTT<br>Reverse: GCTCCATGAGAACACTAGAG
IL-6	Forward: GCCAGAGTCCTTCAGAGAGA<br>Reverse: GGTCTTGGTCCTTAGCCACT
IL-8	Forward: CATCCAGAGCTTGAGTGTGA<br>Reverse: GTTAGCCTTGCCTTTGTTCA
MCP-1	Forward: CATCCAGAGCTTGAGTGTGA<br>Reverse: GTTAGCCTTGCCTTTGTTCA
TSLP	Forward: GGAGATTTGAAAGGGGCTAA<br>Reverse: CATTTCCTGAGTACCGTCAT
S100A8	Forward: CATGCCCTCTACAAGAATGA<br>Reverse: ACCCACTTTTATCACCATCG
GAPDH	Forward: GATGACATCAAGAAGGTGGT<br>Reverse: TACCAGGAAATGAGCTTGAC
